# Pharmacophore and Molecular Docking Guided 3D-QSAR Study of Bacterial Enoyl-ACP Reductase (FabI) Inhibitors

**DOI:** 10.3390/ijms13066620

**Published:** 2012-03-20

**Authors:** Xiaoyun Lu, Man Lv, Kun Huang, Ke Ding, Qidong You

**Affiliations:** 1 Key Laboratory of Regenerative Biology and Institute of Chemical Biology, Guangzhou Institutes of Biomedicine and Health, Chinese Academy of Sciences, No. 190, Kaiyuan Avenue, Science Park, Guangzhou 510530, China; E-Mails: lv_man@gibh.ac.cn (M.L.); ding_ke@gibh.ac.cn (K.D.); 2 Department of Medicinal Chemistry, China Pharmaceutical University, 24 Tongjiaxiang, Nanjing 210009, China; E-Mails: huangkun0214888@yahoo.com.cn (K.H.); youqidong@gmail.com (Q.Y.)

**Keywords:** FabI inhibitors, pharmacophore, molecular docking, 3D-QSAR, CoMFA, CoMSIA

## Abstract

Enoyl acyl carrier protein (ACP) reductase (FabI) is a potential target for the development of antibacterial agents. Three-dimensional quantitative structure-activity relationships (3D-QSAR) for substituted formamides series of FabI inhibitors were investigated using comparative molecular field analysis (CoMFA) and comparative molecular similarity indices analysis (CoMSIA) techniques. Pharmacophore and molecular docking methods were used for construction of the molecular alignments. A training set of 36 compounds was performed to create the 3D-QSAR models and their external predictivity was proven using a test set of 11 compounds. Graphical interpretation of the results revealed important structural features of the formamides related to the active site of FabI. The results may be exploited for further optimization of the design of new potent FabI inhibitors.

## 1. Introduction

The emergence of bacterial resistance to most of the antibiotics currently in clinical use is of world-wide concern [1,2]. In particular, methicillin-resistant *Staphylococcus aureus* (MRSA) [3] and penicillin-resistant *Streptococcus pneumoniae* (PRSP) [4] have become troublesome due to the ineffectiveness of remaining therapeutics. Recently, very few novel classes of antibacterial agents have been marketed. Thus, it is urgently desired to develop antibacterial agents with novel mechanism of action against resistant strains.

As fatty acid biosynthesis in pathogenic microorganisms is essential for cell viability, the enzymes involved in the FAS pathway have recently attracted considerable interest as a genomics-driven target for antibacterial drug discovery [5–7]. The NADH-dependent enoyl acyl carrier protein reductase (FabI) is a key enzyme in the last step of each cycle of fatty acids elongation [8]. It catalyzes the NADH-dependent stereospecific reduction of α,β-unsaturated fatty acids bound to the acyl carrier protein [9,10]. FabI has been identified to be essential for bacterial viability [8]. In recent years, a wide range of structural classes has been identified as FabI inhibitors [11,12], such as triclosan [13–16], diazaborines [17,18], imidazoles [19], indole naphthyridinones [20–22], thiopyridine [23] and 4-pyridone [24], *etc.*, which demonstrates that FabI is a valid target for antibacterial therapy. Based on indole naphthyridinones, further chemical optimization studies and structure activity relationship studies led to the identification of spiro-naphthyridinone piperidines [25] and pyridodiazepines [26] with improved efficacy and desired physiochemical properties. These inhibitors have shown promising results in the preclinical testing in various resistant strains and mouse models. Despite these and other known inhibitors, more structurally diverse inhibitors of FabI need to be discovered for improving the understanding of the biological function of FabI.

With a view to identify potential compounds with higher predicted potencies, novel FabI inhibitors could be designed by virtual screening and molecular docking based on the available X-ray crystal structures of FabI [19,21,22]. However, unlike quantitative structure-activity relationship (QSAR) techniques, these methods do not readily yield information regarding the importance of the molecular substructures for activity. Comparative molecular field analysis (CoMFA) [27] and comparative molecular similarity indices analysis (CoMSIA) [28] are the most popular three-dimensional quantitative structure-activity relationship (3D-QSAR) methods. Their graphic results can provide a direct way to visualize the structure-activity relationships. To the best of our knowledge, no QSAR studies have been reported by now for those FabI inhibitors. In order to gain further insights into the structural and chemical features required for FabI inhibitory activity, we performed 3D-QSAR analyses of a series of formamides published in the literature [21,22,25,26]. An important challenge for the investigated compounds is to deal with their flexibility, since the applications of CoMFA and CoMSIA require the optimized 3D conformations of all molecules. Alignments of ligands that are known to assume the bioactive conformation were generated by superimposition on pharmacophore points and docking into the protein binding site. The 3D-QSAR results provided useful information about the structural requirements for FabI inhibitors and are expected to usefully support drug design of new FabI inhibitors.

## 2. Material and Methods

### 2.1. Data Sets

A dataset comprising 47 substituted formamides was taken from the literature [21,22,25,26]. The biological data were considered comparable and divided into a training set and a test set as shown in Table 1. Thirty-six compounds were selected randomly as the training set for model construction and the remaining 11 (asterisked in Table 1) were used as test set for model validation, according to biological activity range and structural diversity. All further studies were performed using the same training and test sets. The IC_50_ values were converted into pIC_50_ (−logIC_50_) for use in 3D-QSAR analysis.

### 2.2. Pharmacophore Model Generation

The crystal structure of *E. coli* FabI with compound **20** (PDB code: 1MFP) was used as starting structure for the generation of the pharmacophore model. The software LigandScout 3.01 [29,30] was used for detection and interpretation of crucial interaction patterns between FabI and the ligand. LigandScout extracts and interprets ligands and their macromolecular environment from PDB files and automatically creates and visualizes an advanced pharmacophore model. Then the pharmacophore model was exported as a hypoedit script and converted into Discovery Studio 2.1 [31] format with Hypoedit tool. Subsequently, the pharmacophore model was used for mapping all of the molecules.

### 2.3. Molecular Docking

The docking procedure aims to generate and score putative protein-ligand complexes according to their calculated binding affinities. Docking studies were carried out using GOLD docking software [32], version 3.1, which uses a powerful genetic algorithm (GA) method for conformation search and docking, and is widely regarded as one of the best docking programs [33]. Docking experiments were performed using the default GOLD fitness function (VDW = 4.0, H-bonding = 2.5) and default evolutionary parameters: population size = 100; selection pressure = 1.1; operations = 100,000; islands = 5; niche size = 2; migration = 10; mutation = 95; crossover = 95. The ChemScore function was used to rank different binding poses. The center of the bound ligand was defined as the binding site. Ten docking runs were performed per structure. All poses were output into a single *.sdf file.

### 2.4. Alignment Rule

In the 3D-QSAR studies, the molecular alignment and conformation determination are very important to construct reliable models. Due to the flexibility of the investigated compounds, it is difficult to choose a suitable conformation that achieves a meaningful superimposition. In an ideal alignment the biologically active conformations should be aligned taking into account the orientations that the ligands adopt at the binding site of the protein. Therefore, we applied two different receptor-based alignments, with the conformations obtained from structure-based pharmacophore (SBP) search and docking.

All the molecules in the training and test sets were mapped simultaneously onto the pharmacophore model using the “flexible” fitting method and “best mapping only” option in the Ligand Pharmacophore Mapping protocol in Discovery Studio 2.1. The conformation selected for each compound, which was assumed to be the bioactive conformation, corresponded to the conformation which best fit the pharmacophore model. The final aligned molecules were exported to SYBYL6.9 [34] for CoMFA and CoMSIA analysis.

For the docking, all the molecules were docked into the FabI active site using the GOLD program. The conformation with the highest ChemScore of each molecule and their alignment were used directly in CoMFA and CoMSIA to explore 3D-QSAR models.

### 2.5. CoMFA and CoMSIA Model

CoMFA was performed using the QSAR option of SYBYL 6.9. The steric and electrostatic field energies were calculated using the Lennard-Jones and the Coulomb potentials, respectively, with a 1/r distance-dependent dielectric constant in all intersections of a regularly spaced (0.2 nm) grid. The electrostatic fields were computed using Gasteiger-Huckel charge calculation methods. A sp3 hybridized carbon atom with a radius of 1.53 Å and a charge of +1.0 was used as a probe to calculate the steric and electrostatic energies between the probe and the molecules using the Tripos force field. The standard parameters implemented in SYBYL 6.9 were used. The truncation for both the steric and the electrostatic energies were set to 30 kcal/mol.

The CoMSIA method involves a common probe atom and similarity indices calculated at regularly spaced grid intervals for the pre-aligned molecules. The similarity indices descriptors were derived with the same lattice box used in CoMFA. CoMSIA calculates hydrophobic, H-bond donor and acceptor fields in addition to steric and electrostatic fields. The distance dependence between the grid point and each atom of molecule was determined by the Gaussian function through the similarity indices calculated at all grid points, and a default value of 0.3 was used as an attenuation factor.

### 2.6. Partial Least Square Analysis

Partial least squares (PLS) [35] methodology was used for all the 3D-QSAR analyses. The cross-validation [36,37] analysis was performed using the leave one out (LOO) method in which one compound is removed from the dataset and its activity is predicted using the model derived from the rest of the dataset. The cross validated *r*^2^ that resulted in the optimum number of components and lowest standard error of prediction were considered for further analysis. To speed up the analysis and reduce noise, a minimum filter value σ of 2.00 kcal/mol was used. Final analysis was performed to calculate conventional *r*^2^ using the optimum number of components obtained from the cross-validation analysis.

The predictive power of the 3D-QSAR models was determined from a set of eight molecules that were excluded during model development. The optimization, alignment and all other steps of these test set molecules were the same as those of the training set molecules described above, and their activities were predicted using the model produced by the training set. The predictive correlation (*r*_pred_^2^) based on the test set molecules, is computed using

$${r_{\text{pred}}}^{2}\;=(\text{SD}-\text{PRESS})/\text{SD}$$

where, SD is defined as the sum of the squared deviations between the biological activities of the test set and mean activity of the training set molecules and PRESS is the sum of the squared deviation between the predicted and actual activity values for each molecule in the test set.

## 3. Results and Discussion

### 3.1. Pharmacophore-based CoMFA and CoMSIA

#### 3.1.1. Pharmacophore Elucidation

**Figure 1:**
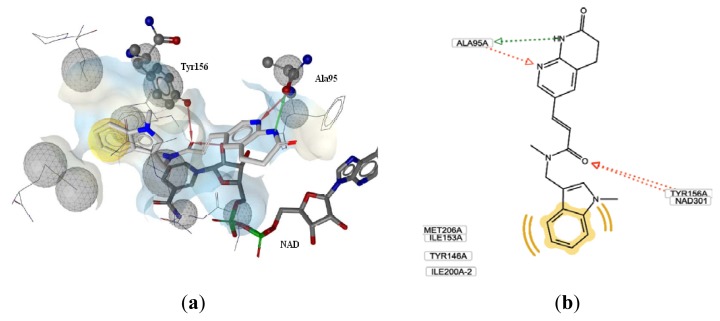
(**a**) LigandScout pharmacophore model generated from FabI-compound **20** crystal structure (red arrows, HBA; green arrow, HBD; yellow spheres, hydrophobic sites; gray spheres, excluded volumes); (**b**) Schematic 2D molecular interactions plot of compound **20** with residues of the FabI binding site.

As shown in Figure 1a, the pharmacophore model automatically generated by the LigandScout 3.01 program includes five features: three hydrogen bond acceptors (HBA), one hydrogen bond donor (HBD) and one hydrophobic group. Besides, the program automatically generated a series of excluded volumes in the model. Two HBA features characterize the carbonyl group of the ligand, which forms two hydrogen bonds with Tyr156 and the 2′-hydroxyl group of the nicotinamide ribose of the nucleotide (Figure 1b). The other HBA and the HBD features are involved in H-bond interactions between alanine 95 and the pyridylamine and *N*-acyl hydrogen of the naphthyridinone functionality, respectively. The hydrophobic feature is located on the indole portion.

**Figure 2:**
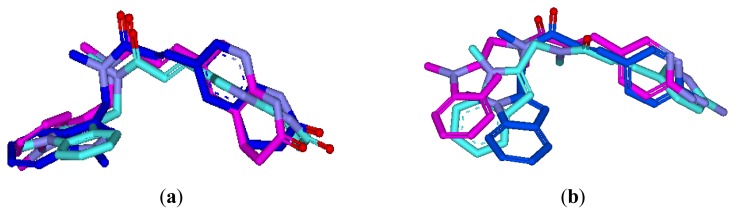
The best pharmacophore mapping conformation (purple stick) and the docking conformation with the highest ChemScore (cyan stick) are superimposed on the bound conformation in the crystal structure (blue stick). (**a**) For compound **20**; (**b**) for compound **9**.

**Figure 3:**
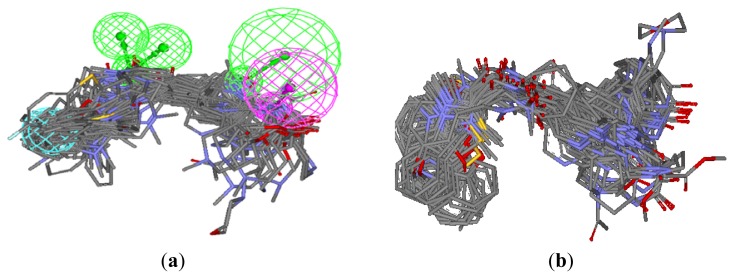
(**a**) Alignment of the training and test set molecules based on the pharmacophore model; (**b**) docking-based alignment of the training and test set molecules.

To verify whether the pharmacophore model finds the correct bioactive conformation, we applied the method to two structurally similar compounds **20** and **9**, whose bioactive conformations are known from *E. coli* X-ray structures of the ligand-enzyme complex. Their bound conformations were mapped onto the pharmacophore model using the “flexible” fitting method and “best mapping only” option in the Ligand Pharmacophore Mapping protocol and then superimposed on the best mapping conformations (Figure 2). From the results, it is important to note that the best mapping conformations of the ligands are in agreement with their bioactive conformations. Hence, the results showed that the pharmacophore model is capable of reproducing the bioactive conformation from the Protein Data Bank and is reliable enough to retrieve compounds that fit all the features of the query from chemical databases. The final aligned molecules are shown in Figure 3a.

#### 3.1.2. CoMFA and CoMSIA Results

The stepwise development of CoMFA and CoMSIA models using different fields is presented in Table 2. The CoMFA model leads to a *r*_cv_^2^ value of 0.668 using four components and non-cross validated *r*^2^ = 0.980 with SEE = 0.213 and *F* = 371.89. These values indicate that the CoMFA model has a good conventional statistical correlation and it allows good predictions of the biological activity data of formamides also for the test set. The CoMSIA was performed using steric, electrostatic, hydrophobic and hydrogen-bond donor and acceptor descriptors. As shown in Table 2, a combination of steric, electrostatic, hydrophobic and hydrogen-bond donor resulted in a CoMSIA model with a higher *r*_cv_^2^ value of 0.742, a good *r*^2^ value of 0.973, compared to the other CoMSIA model. Therefore, it was selected as the best model to generate contour maps and explain the SAR.

**Figure 4:**
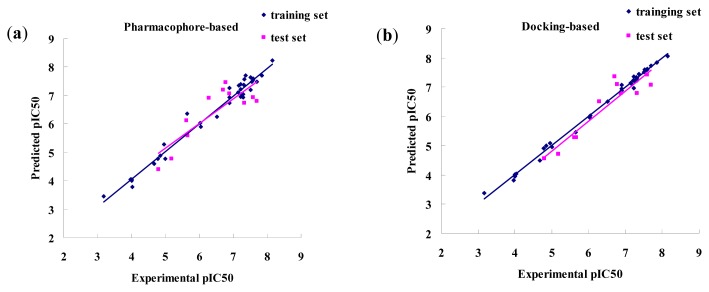
Graph of experimental values versus predicted values for training and test set compounds from CoMSIA-SEHD model. (**a**) For pharmacophore-based alignment, (**b**) for docking-based alignment.

The external validation is important to establish a reliable 3D-QSAR model. To validate the stability and predictivity of the 3D-QSAR models, 11 compounds that were not included in the construction of the 3D-QSAR models were selected as the test set. The CoMSIA-SEHD model showed the best reasonable external predictivity, yielding a *r*_pred_^2^ of 0.702 (Table 2). The predicted values of training and test sets are presented in Table 3. The correlations between the predicted and experimental values of all compounds are shown in Figure 4a.

### 3.2. Molecular Docking-Based CoMFA and CoMSIA

#### 3.2.1. Docking Analysis

To test whether the GOLD program is feasible for ligand binding to FabI, two X-ray crystal structures (PDB code, 1FMP [22] and 1LXC [21]) were initially chosen and the docked conformation of ligands was compared with their crystallographic conformation. The proteins were prepared by removing the small ligands and adding hydrogens using SYBYL 6.9. The active site for docking was defined as all atoms within 8 Å radius of the co-crystallized ligand. The resulting docked conformation with highest ChemScore value and that of crystallographic conformation were very similar (Figure 2). This result demonstrates that GOLD analysis is suitable for the identification of the binding mode between inhibitors and FabI. The resulting alignment of all compounds is shown in Figure 3b. This molecular alignment is more realistic and more suitable for molecular field analyses.

#### 3.2.2. CoMFA and CoMSIA Results

The CoMFA and CoMSIA results generated from docked conformations are presented in Table 4. The CoMFA models showed *r*_cv_^2^ of 0.664 with six components, *r*^2^ of 0.993 and *F* value of 730.83. For CoMSIA, differing from the pharmacophore alignment, the best *r*_cv_^2^ was obtained using only steric and electrostatic descriptor variables. The high value of *r*_cv_^2^ appears to be a necessary but not the sufficient condition for the model to have a high predictive power. A CoMSIA-SEHD model with a *r*_cv_^2^ of 0.711 with six components, a *r*^2^ of 0.995 and a best external predictivity *r*_pred_^2^ of 0.864 was obtained. These data demonstrate that a more statistically robust model was obtained from the CoMSIA study. The experimental versus predicted activities (Table 4) using CoMSIA-SEHD models of all compounds are shown in Figure 4b.

### 3.3. CoMSIA Models Interpretation

**Figure 5:**
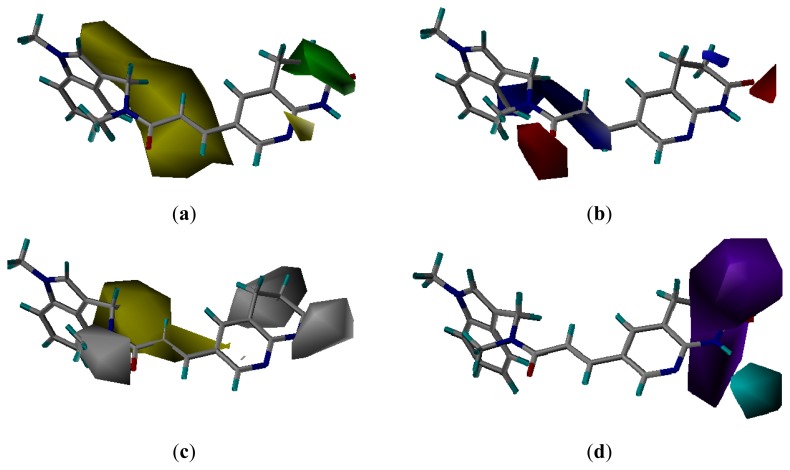
CoMSIA contour maps from the pharmacophore-based alignment. (**a**) CoMSIA steric and electrostatic contour map. Green and yellow represent sterically favored and disfavored regions, respectively. (**b**) CoMSIA electrostatic contour map. Blue and red represent electrically favored and disfavored regions, respectively. (**c**) CoMSIA hydrophobic field contour map. Yellow regions indicate where hydrophobic groups increased activity and white regions indicate areas where hydrophilic groups increased activity. (**d**) CoMSIA H-bond donor contour map. Cyan contour indicates regions where hydrogen bond donor groups increased activity. Purple is disfavored. Compound **20** is shown inside the fields.

**Figure 6:**
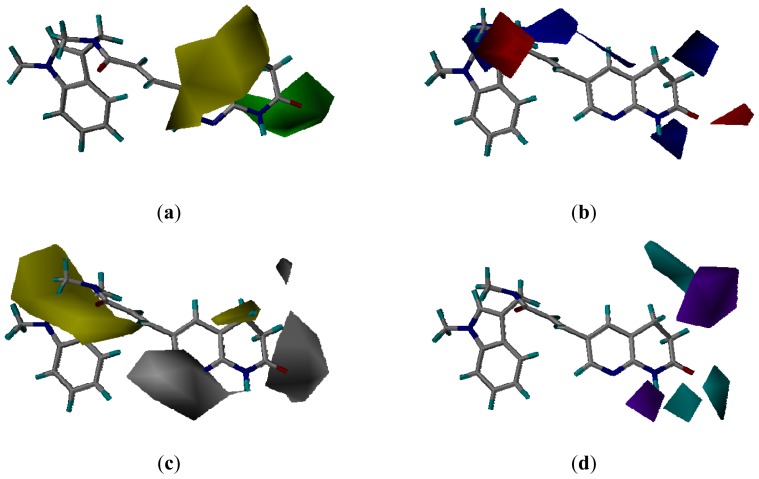
CoMSIA contour maps from docking-based alignment. (**a**) CoMSIA steric and electrostatic contour map. Green and yellow represent sterically favored and disfavored regions, respectively. (**b**) CoMSIA electrostatic contour map. Blue and red represent electrically favored and disfavored regions, respectively. (**c**) CoMSIA hydrophobic field contour map. Yellow regions indicate where hydrophobic groups increased activity and white regions indicate areas where hydrophilic groups increased activity. (**d**) CoMSIA H-bond donor contour map. Cyan contour indicates regions where hydrogen bond donor groups increased activity. Purple is disfavored. Compound **20** is shown inside the fields.

Steric, electrostatic, hydrophobic and hydrogen bond donor contour plots obtained using CoMSIA analysis from pharmacophore and docking based alignment are useful to explore the protein-ligand interactions, presented in Figures 5 and 6, respectively. For simplicity, the interactions between only compound **20** and the contour plots are shown.

#### 3.3.1. Pharmacophore-Based Alignment

As shown in Figure 5a, the green contour observed above the naphthyridinone ring indicated that some bulky substitutions at this region would be favorable for the activity, which was known to be exposed to solvent. This can explain the fact that compounds **26**–**33** with spiropiperidine substitutions and compounds **34**–**47** with seven- and eight-membered heterocyclic rings exhibited potent FabI inhibitory activity. A large yellow contour found around the ene-amide of compound **20** suggested that any bulky substitution at this position would be likely to decrease activity. The orientation of the linking amide may be very important for activity.

The electrostatic contour map is shown as red and blue contours in Figure 5b. The small blue contour near the naphthyridinone ring suggested that the presence of positively charged groups in this region was favorable for activity. Besides, a large positively charged blue contour was found near ene-amide too. Two carbonyls of compound **20** were oriented towards two red contours, thereby indicating favorable interactions with negatively charged groups.

The hydrophobic contour map of CoMSIA is presented in Figure 5c. The hydrophilic favored white contour around the naphthyridinone ring implied that any hydrophilic substitution was favored there. This was in accordance with compounds **25**–**33**, where incorporating hydrophilic morpholine or piperidine proved to improve the activity. The yellow contour indicated that any hydrophobic group substituent here was favored. This region supported the observation that compounds **29** and **33**, with OPr substitution, were among the compounds with a high FabI inhibitory activity.

The hydrogen bond donor contour plot is displayed in Figure 5d. The cyan contours represent the region favoring hydrogen bond donor substituents. The purple contours indicate that hydrogen bond acceptor groups in these regions are required for higher activity. It can be explained by the fact that the hydrogen bonds were involved in the naphthyridinone functionality and enzyme FabI. That was why compounds **1**–**16** without naphthyridinone were less potent compared to compounds **19**–**24**, *etc*. For compounds **17** and **18**, acylated amino pyridine was less favored compared to the hydrogen bond of naphthyridinone.

#### 3.3.2. Molecular Docking-Based Alignment

The CoMSIA contour plots from the docking-based alignment are shown in Figure 6. The steric, electrostatic and hydrophobic contour maps of CoMSIA were similar to those of CoMSIA from the pharmacophore-based alignment in explaining the effect of substitution on the biological activity (Figure 6a–c). One difference was that a yellow region around the pyridine ring represented an area where no substitution was favored.

For the hydrogen bond donor contour map, the two small cycan contours around the *N*-acylhydrogen indicated that hydrogen bond donor substituents would improve the activity. This can be explained by the fact that compounds **17**–**47**, which possessed *N*-acylhydrogen, showed better potencies than compounds **1**–**16**. The other cycan contour above the naphthyridinone revealed that hydrogen bond donor substituents might be favored. This may be the reason why a series of seven- and eight-membered heterocyclic derivatives **34**–**39** and **41**–**47** bearing a free NH atom showed improved potencies compared to compound **40** with nitrogen methylation. A small purple contour at the nitrogen position of pyridine suggested that a hydrogen bond acceptor substituent at this position might be favored. Most of the active derivatives (**11**, **12**, **14**–**47**) possessed hydrogen bond acceptor groups such as a nitrogen atom at this position. Compounds **7**–**10** and **13** without a hydrogen bond acceptor substituent at this position exhibited significantly decreased potencies. Another purple contour around the naphthyridinone also revealed that a hydrogen bond acceptor group would improve the activity. This was consistent with the fact that compounds **45** and **46** bearing the carbonyl group at this position exhibited a higher activity than compound **47**.

Despite the different alignments, their 3D-QSAR models are comparable to those presented there. However, this docking-based study provides significantly better predicted statistical coefficients (*r*_pred_^2^ = 0.864) and additional information about the hydrogen bond interactions of the naphthyridinone moiety, which emphasizes the benefit of combining docking for the selection of the alignment followed by 3D-QSAR for formamides-based FabI inhibitors.

### 3.4. Summary of Structure-Activity Relationships

**Figure 7:**
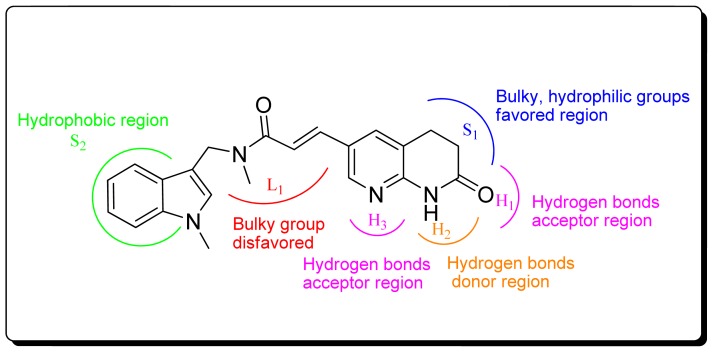
Structural requirements for formamides based FabI inhibitors.

Comparing the 3D-QSAR models, the improved performance of the CoMSIA is obvious, while the differences in the case of CoMFA are negligible. The results of 3D-QSAR CoMSIA obtained from both alignments are complimentary to each other. The detailed contour analysis of both CoMSIA models enabled us to point out several structural requirements as mentioned in the above discussion for the observed inhibitory activities (Figure 7). In detail, the bulky and hydrophilic groups at the S_1_ region are favorable, while the hydrophobic groups at the S_2_ region are favorable. The hydrogen bond acceptor group at the H_1_ and H_3_ position may benefit the potency. The hydrogen bond donor group at the H_2_ position increases the activity. The ene-formamide skeleton was essential for high activity.

## 4. Conclusions

In this study, the conformations of all compounds obtained from the pharmacophore-based alignment and docking into the active site of FabI were used for the CoMFA and CoMSIA analyses. Both alignment procedures led to statistically robust and predictive 3D-QSAR models. Despite the different alignments, the contour maps from both models are similar in explaining the influence of substitution on activity. The predictive ability of the models was confirmed by predicting the activity of 11 compounds used as external test sets. The robust 3D-QSAR model and its three-dimensional contour map provide guidelines to design compounds with new scaffolds and optimize known molecules.

## Figures and Tables

**Table 1:** Structures, activities and experimental pIC_50_ values of the compounds in the training and test sets. The test set compounds are marked by an asterisk *.

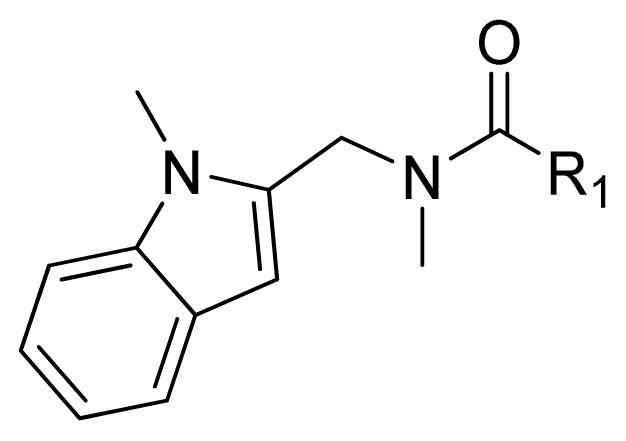				

Compounds	R_1_	IC_50_(μM)	pIC_50_	
1	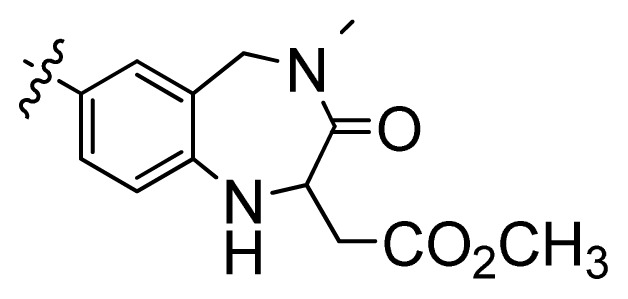	16.5	4.78	
2	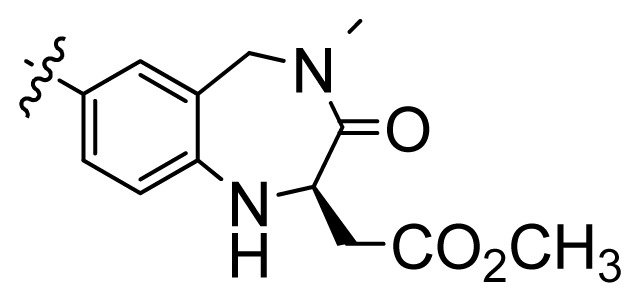	9.9	5.00	
3	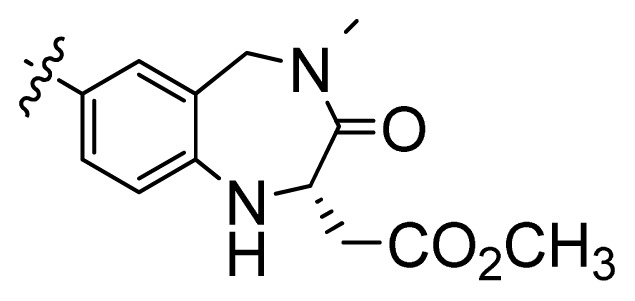	676	3.17	
4	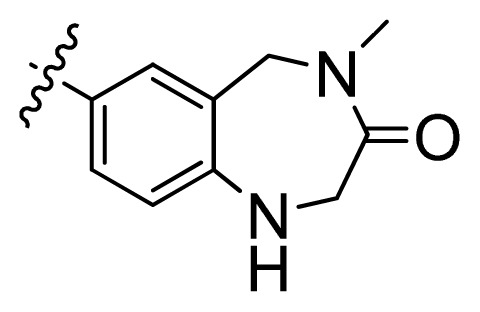	21.2	4.67	
5 *	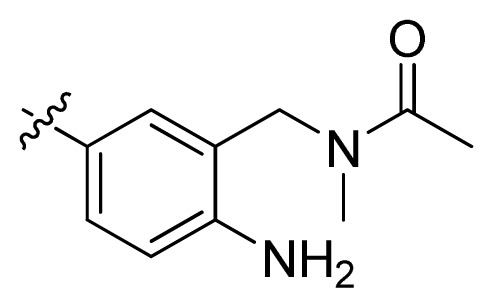	6.7	5.17	
6	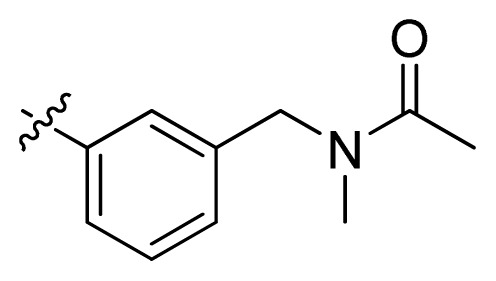	107	3.97	
7 *	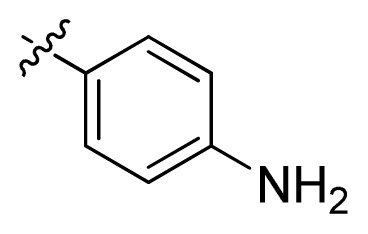	16.3	4.79	
8	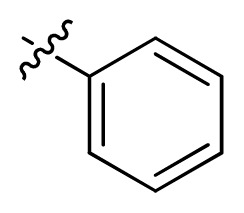	100	4.00	
9	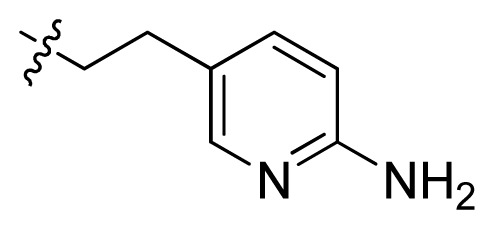	93.7	4.03	
10	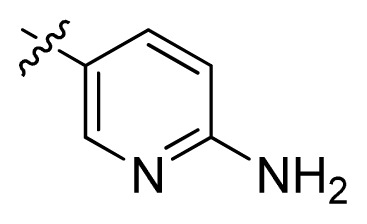	100	4.00	

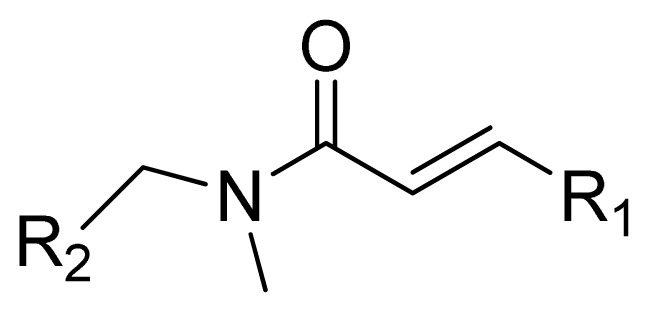				

Compounds	R_1_	IC_50_(μM)	pIC_50_	Compounds

11 *	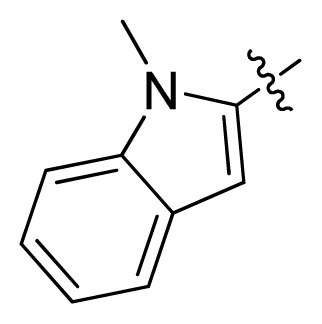	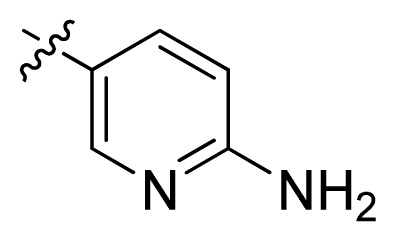	2.4	5.62
12 *	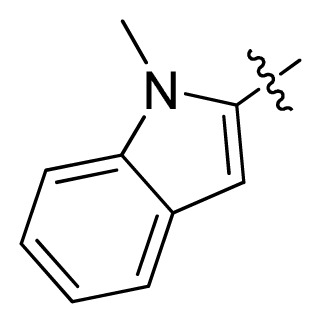	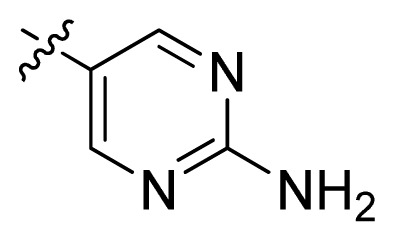	2.2	5.66
13	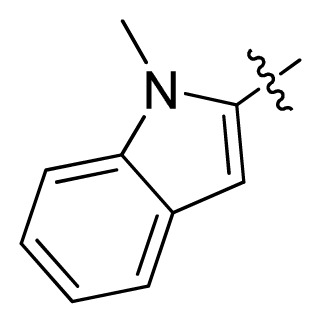	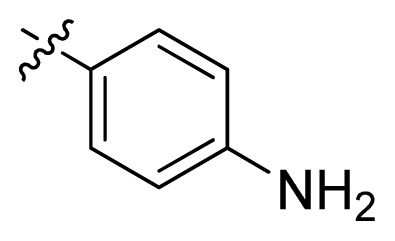	11.2	4.95
14	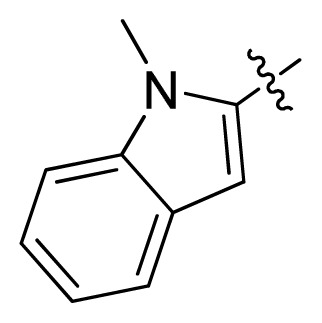	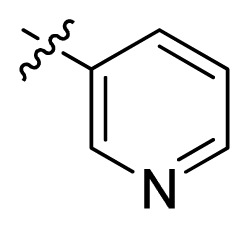	14.2	4.85
15	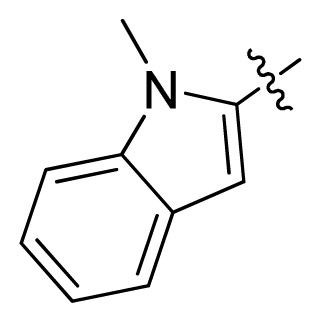	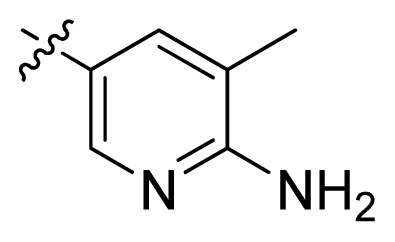	0.91	6.04
16	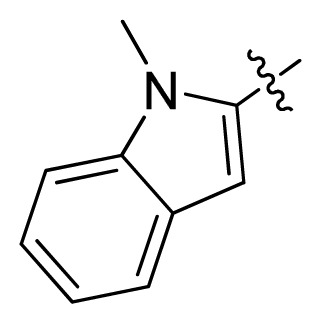	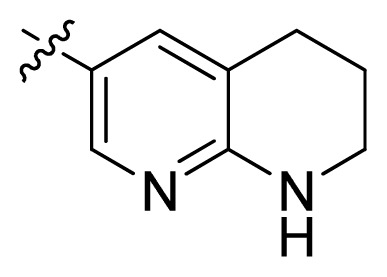	2.3	5.64
17	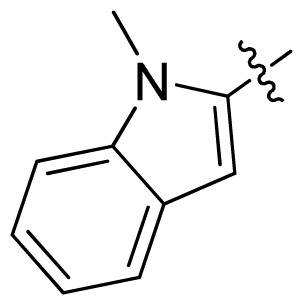	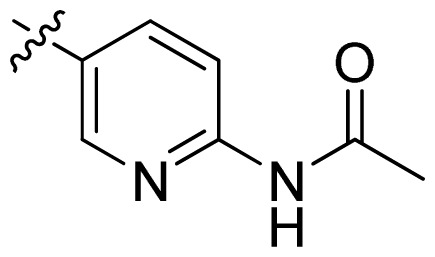	0.3	6.52
18	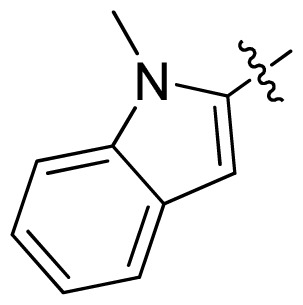	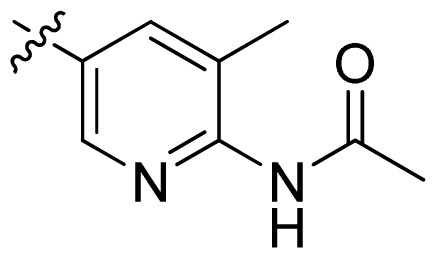	0.94	6.03
19	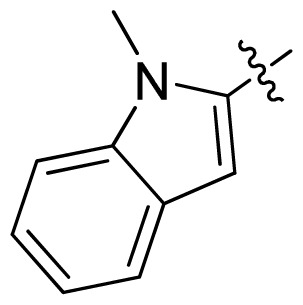	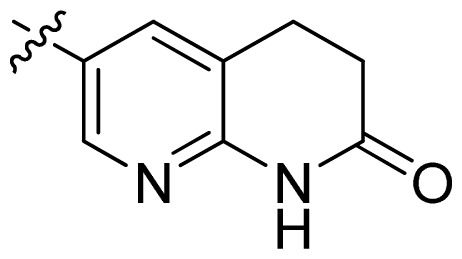	0.05	7.30
20	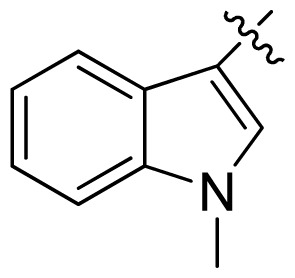	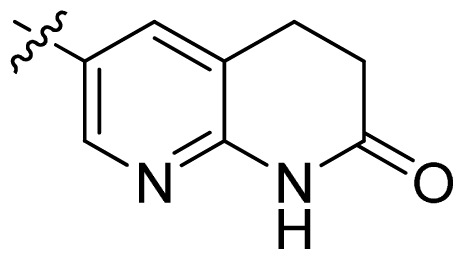	0.13	6.89
21	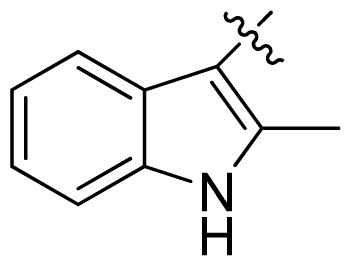	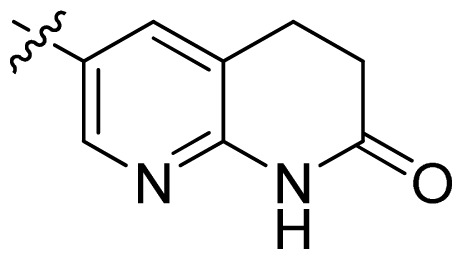	0.05	7.30
22	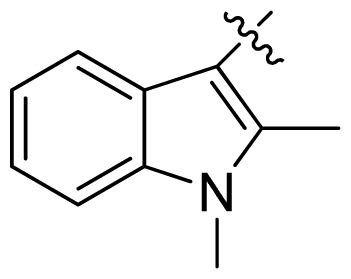	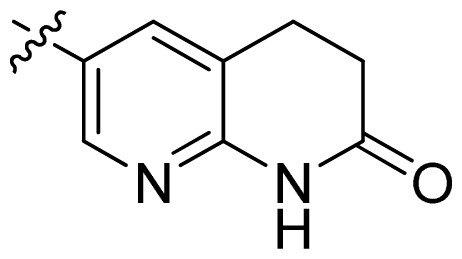	0.06	7.22
23 *	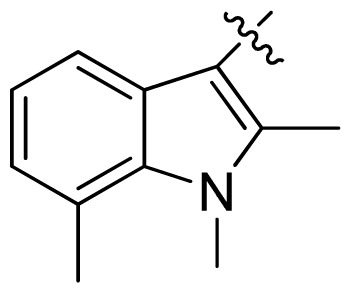	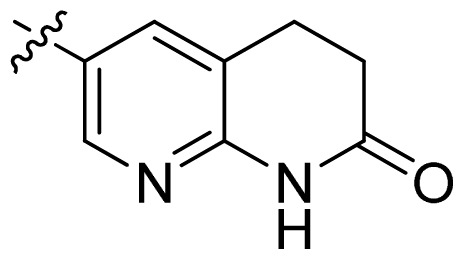	0.02	7.70
24	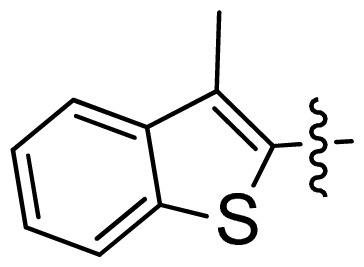	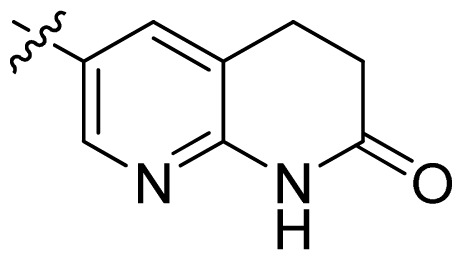	0.03	7.52
25	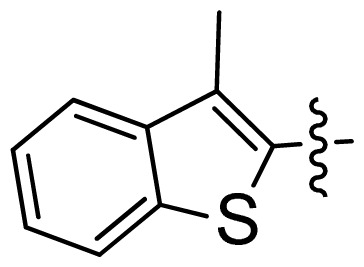	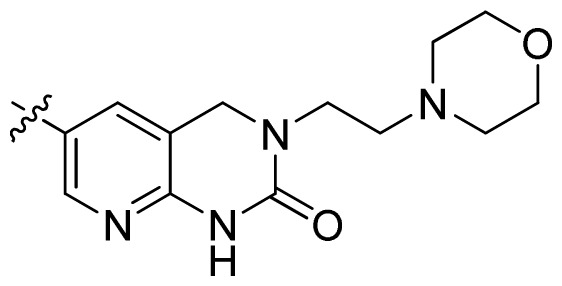	0.026	7.59
26	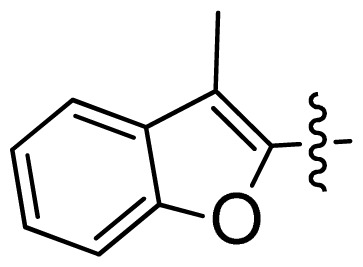	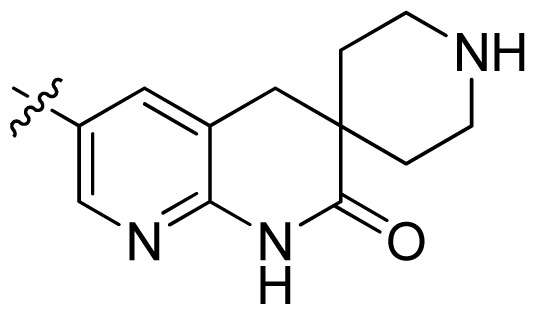	0.049	7.31
27 *	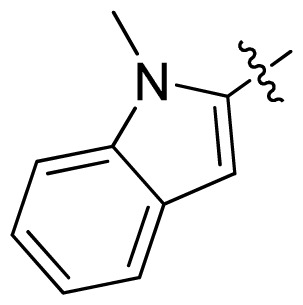	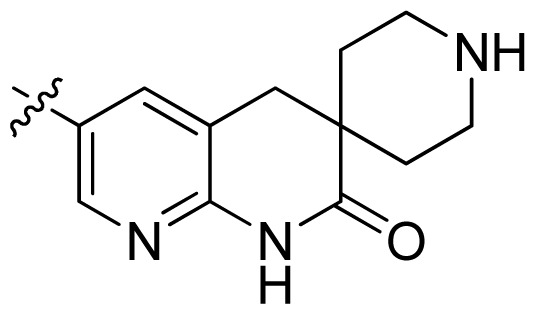	0.132	6.88
28	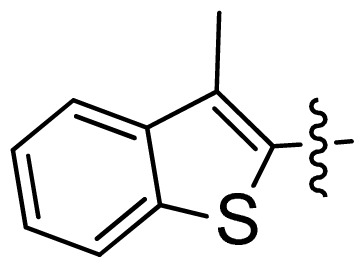	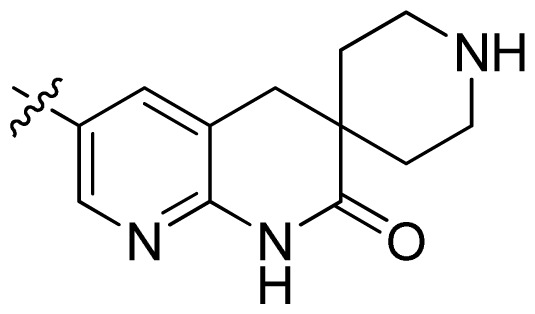	0.048	7.32
29	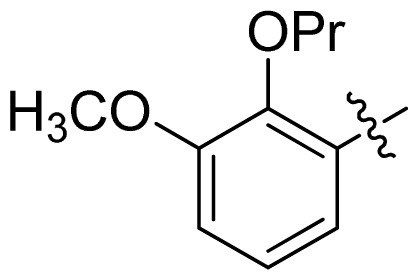	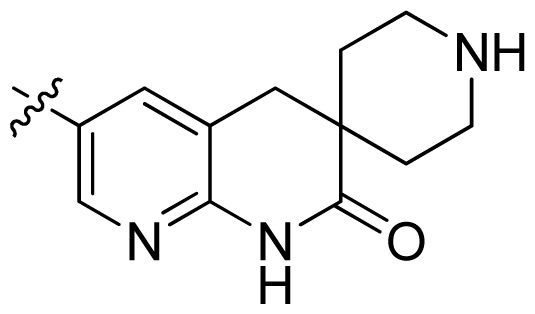	0.071	7.15
30	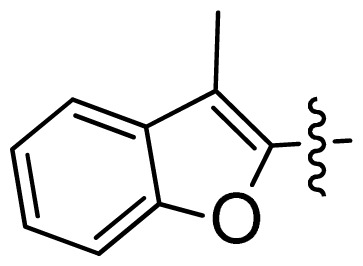	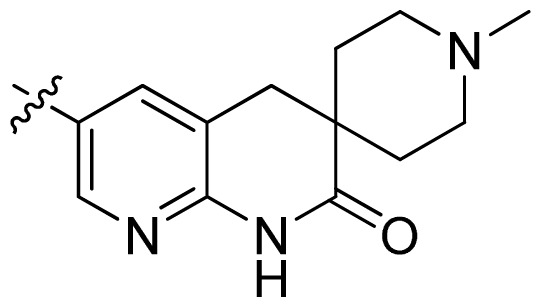	0.02	7.70
31	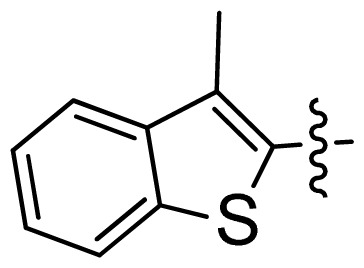	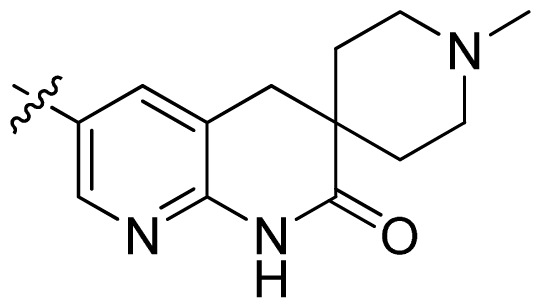	0.014	7.85
32	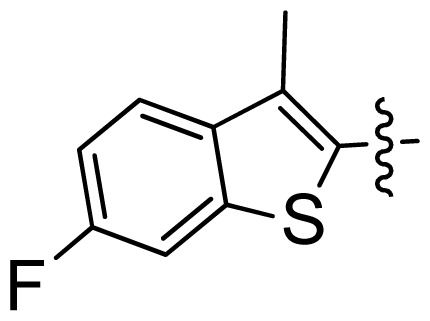	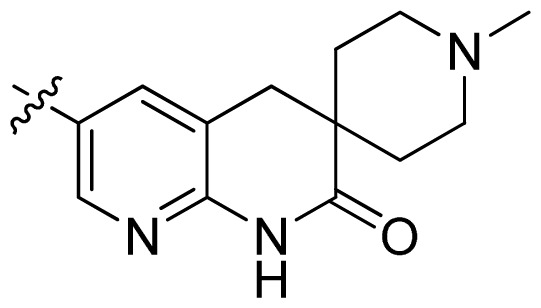	0.028	7.55
33	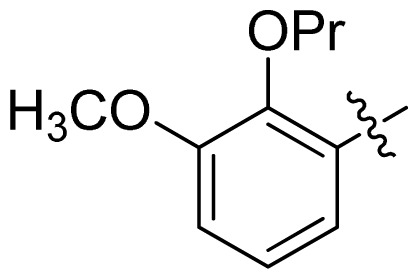	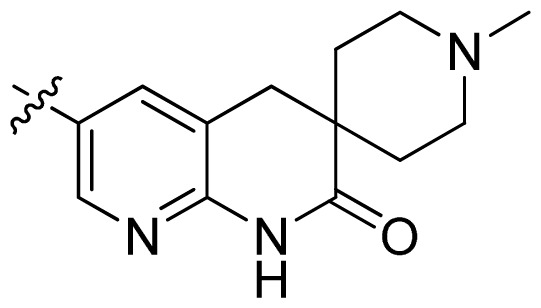	0.026	7.59
34	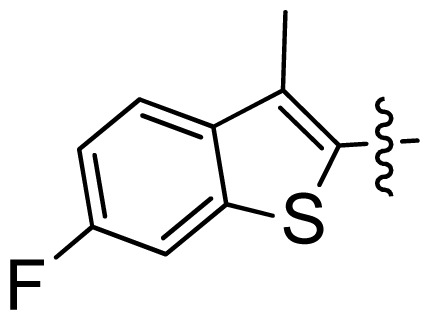	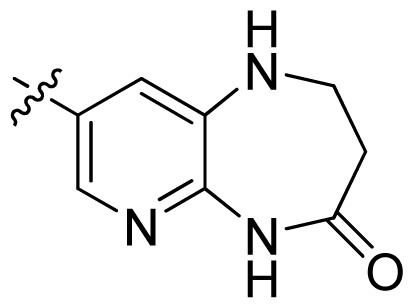	0.031	7.51
35	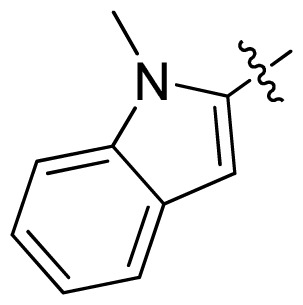	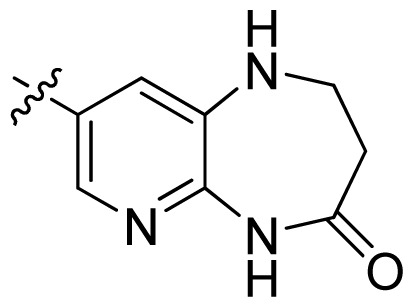	0.13	6.89
36 *	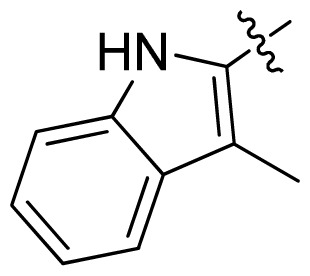	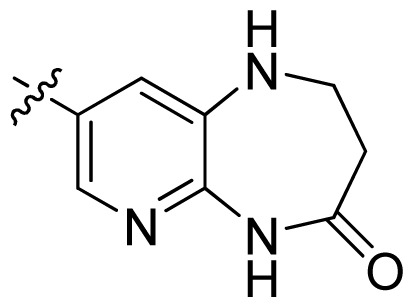	0.2	6.70
37	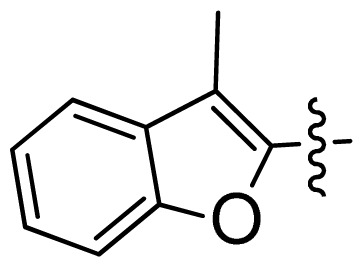	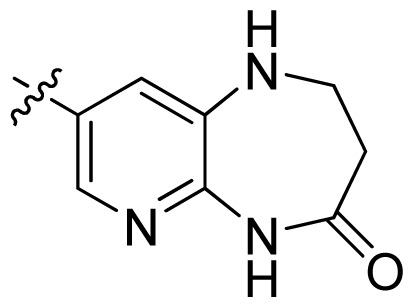	0.007	8.15
38 *	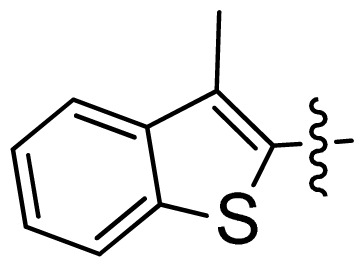	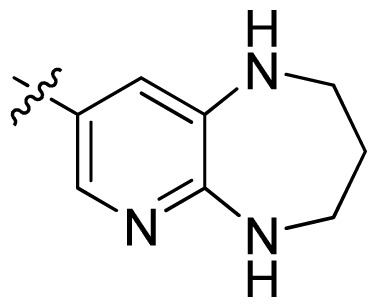	0.026	7.59
39	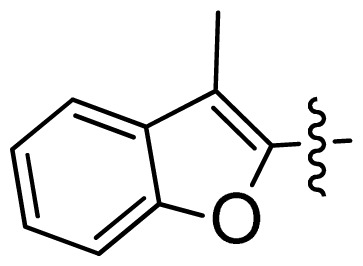	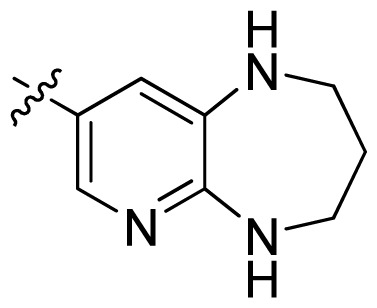	0.067	7.17
40 *	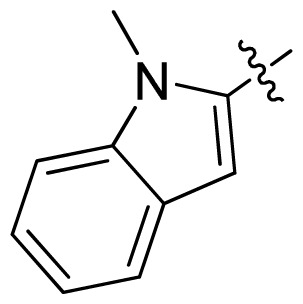	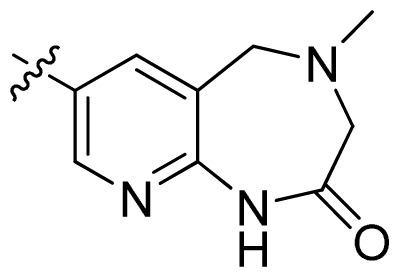	0.51	6.29
41	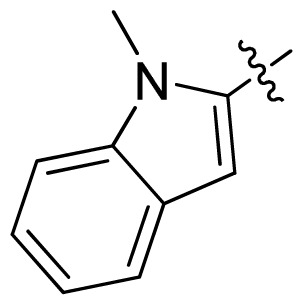	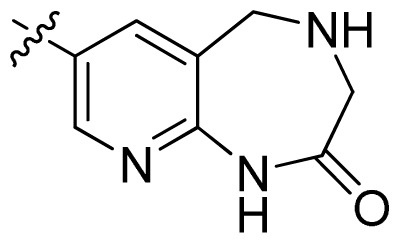	0.13	6.89
42	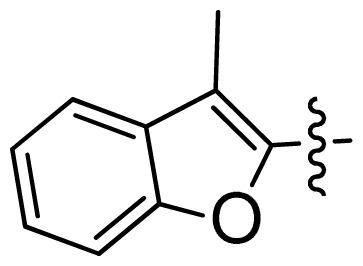	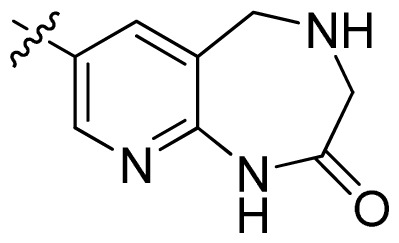	0.043	7.37
43	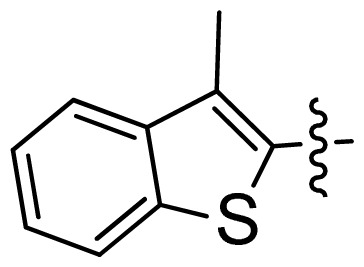	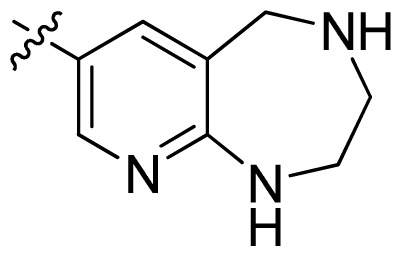	0.057	7.24
44 *	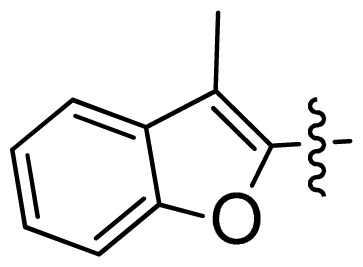	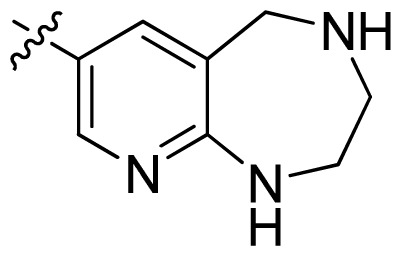	0.048	7.32
45	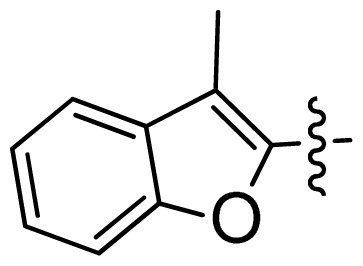	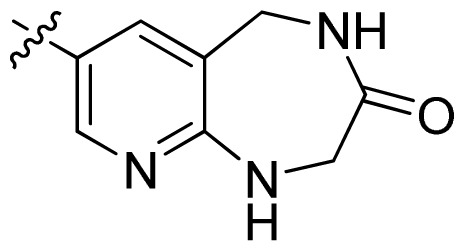	0.06	7.22
46	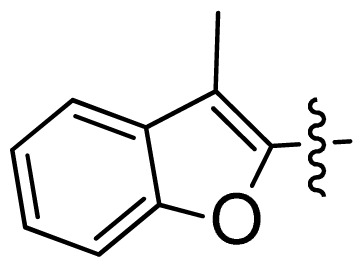	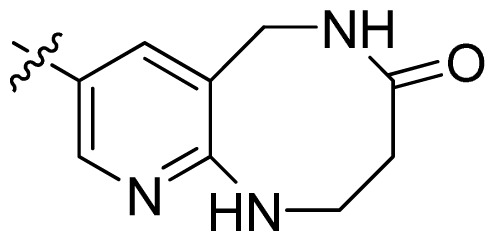	0.061	7.21
47 *	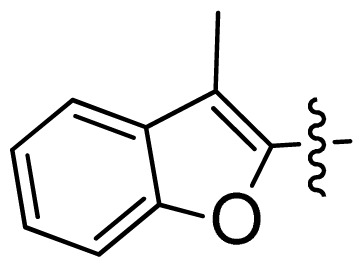	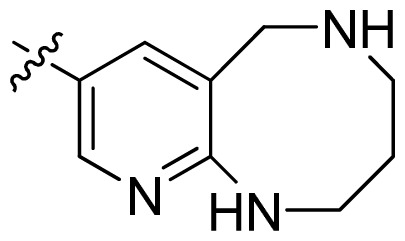	0.17	6.77

**Table 2:** Results of CoMFA and CoMSIA analyses with pharmacophore-based alignments.

Parameter	CoMFA	CoMSIA								

	S,E	S	E	H	D	A	S,E	H,D,A	S,E,H,D	ALL
*r*_cv_^2^	0.668	0.478	0.580	0.554	0.382	0.565	0.700	0.665	0.742	0.721
*N*	4	4	2	3	2	5	4	6	3	4
*r*^2^	0.980	0.895	0.856	0.944	0.727	0.910	0.973	0.996	0.973	0.988
SEE	0.213	0.481	0.547	0.345	0.754	0.454	0.243	0.093	0.240	0.162
*F*-value	371.89	66.39	98.12	181.23	43.92	60.62	282.57	1313.59	386.54	649.64
*Contributions*										
Steric	0.490						0.374		0.152	0.122
Electrostatic	0.510						0.636		0.277	0.217
Hydrophobic								0.341	0.253	0.208
Donor								0.358	0.318	0.267
Acceptor								0.301		0.187
*r*_pred_^2^	0.574								0.702	0.693

*r*_cv_^2^ = cross-validated correlation coefficient; *N* = No. of components; *r*^2^ = conventional correlation coefficient; SEE = standard error of estimate; *F*-value = *F*-test value; S = steric field; E = electrostatic field; H = hydrophobic field; D = hydrogen bond donor field; A = hydrogen bond acceptor field; *r*_pred_^2^ = predicted correlation coefficient for test set of compounds.

**Table 3:** Experimental, predicted and residuals of pIC_50_ values of investigated compounds in the training and test sets from the best selected three-dimensional quantitative structure-activity relationship (3D-QSAR) models.

Compounds	Experimental Activity	Pharmacophore-Based Alignment				Docking-Based Alignment			

	(pIC_50_)	CoMFA (SE)		CoMSIA (SEHD)		CoMFA (SE)		CoMSIA (SEHD)	

		PA a	Δ b	PA a	Δ b	PA a	Δ b	PA a	Δ b
1	4.78	4.73	0.05	4.77	0.01	4.72	0.06	4.91	−0.13
2	5.00	5.17	−0.17	4.77	0.23	4.91	0.09	4.95	0.05
3	3.17	3.15	0.02	3.46	−0.29	3.26	−0.09	3.37	−0.20
4	4.67	4.45	0.22	4.59	0.08	4.79	−0.12	4.50	0.17
5 *	5.17	5.95	−0.78	4.78	0.39	4.32	0.85	4.71	0.46
6	3.97	3.94	0.03	4.04	−0.07	4.01	−0.04	3.81	0.16
7 *	4.79	4.76	0.03	4.41	0.38	4.91	−0.12	4.56	0.20
8	4.00	4.18	−0.18	4.00	0.00	3.99	0.01	3.95	0.05
9	4.03	4.13	−0.10	3.79	0.24	4.09	−0.06	4.04	−0.01
10	4.00	3.94	0.06	4.04	−0.04	3.87	0.13	4.02	−0.02
11 *	5.62	5.48	0.14	6.12	−0.50	5.01	0.61	5.28	0.34
12 *	5.66	5.70	−0.04	5.58	0.08	5.39	0.31	5.28	0.38
13	4.95	5.00	−0.05	5.28	−0.33	4.87	0.08	5.09	−0.14
14	4.85	4.68	0.17	4.88	−0.03	4.89	−0.04	4.99	−0.14
15	6.04	6.01	0.03	5.90	0.14	5.83	0.21	6.02	0.02
16	5.64	6.24	−0.60	6.35	−0.71	5.84	−0.20	5.45	0.19
17	6.52	6.48	0.04	6.24	0.28	6.44	0.08	6.50	0.02
18	6.03	6.03	0.00	6.02	0.01	6.04	−0.01	5.95	0.08
19	7.30	7.04	0.26	6.92	0.38	7.18	0.12	7.34	−0.04
20	6.89	6.94	−0.05	6.73	0.16	6.97	−0.08	6.94	−0.05
21	7.30	7.22	0.08	7.04	0.26	7.30	0.00	7.27	0.03
22	7.22	7.27	−0.05	7.22	0.00	7.22	0.00	6.96	0.26
23 *	7.70	7.10	0.60	6.80	0.90	6.77	0.93	7.07	0.63
24	7.52	7.23	0.29	7.20	0.32	7.54	−0.02	7.59	−0.07
25	7.59	7.60	−0.01	7.60	−0.01	7.54	0.05	7.62	−0.03
26	7.31	7.30	0.01	7.37	−0.06	7.21	0.10	7.23	0.08
27 *	6.88	6.90	−0.02	7.07	−0.17	7.14	−0.26	6.80	0.08
28	7.32	7.42	−0.10	7.56	−0.24	7.52	−0.20	7.31	0.01
29	7.15	7.01	0.14	7.11	0.04	7.16	−0.01	7.15	0.00
30	7.70	7.91	−0.21	7.48	0.22	7.82	−0.12	7.74	−0.04
31	7.85	7.75	0.10	7.70	0.15	7.74	0.11	7.83	0.02
32	7.55	7.61	−0.06	7.49	0.06	7.57	−0.02	7.58	−0.03
33	7.59	7.57	0.02	7.54	0.05	7.55	0.04	7.59	0.00
34	7.51	7.64	−0.13	7.64	−0.13	7.48	0.03	7.52	−0.01
35	6.89	7.03	−0.14	7.26	−0.37	7.05	−0.16	7.08	−0.19
36 *	6.70	6.78	−0.08	7.20	−0.50	7.49	−0.79	7.37	−0.59
37	8.15	7.63	0.52	8.23	−0.08	7.93	0.22	8.07	0.08
38 *	7.59	6.57	1.02	6.94	0.65	6.63	0.96	7.42	0.17
39	7.17	7.30	−0.13	7.36	−0.19	6.96	0.21	7.09	0.08
40 *	6.29	7.50	−1.21	6.90	−0.61	6.84	−0.55	6.50	−0.21
41	6.89	7.14	−0.25	6.93	−0.04	6.96	−0.07	6.88	0.01
42	7.37	7.58	−0.21	7.71	−0.34	7.46	−0.09	7.44	−0.07
43	7.24	6.81	0.43	7.03	0.21	7.14	0.10	7.21	0.03
44 *	7.32	6.53	0.79	6.73	0.59	7.16	0.16	6.79	0.53
45	7.22	7.32	−0.10	7.39	−0.17	7.42	−0.20	7.36	−0.14
46	7.21	7.15	0.06	6.95	0.26	7.31	−0.10	7.23	−0.02
47 *	6.77	7.02	−0.25	7.47	−0.70	7.10	−0.33	7.10	−0.33

* Test set molecules;

a Predicted activity;

b Residual of experimental and predicted activities.

**Table 4:** Results of CoMFA and CoMSIA analyses with docking-based alignments.

Parameter	CoMFA	CoMSIA								

	S,E	S	E	H	D	A	S,E	H,D,A	S,E,H,D	ALL
*r*_cv_^2^	0.664	0.486	0.740	0.708	0.128	0.540	0.777	0.548	0.711	0.713
*N*	6	5	6	5	2	2	6	4	6	6
*r*^2^	0.993	0.877	0.975	0.974	0.595	0.798	0.987	0.957	0.995	0.995
SEE	0.125	0.531	0.244	0.246	0.919	0.649	0.172	0.309	0.112	0.109
*F*-value	730.83	42.77	186.96	183.83	24.20	65.07	380.31	171.60	913.60	966.44
*Contributions*										
Steric	0.381						0.270		0.142	0.105
Electrostatic	0.619						0.730		0.383	0.279
Hydrophobic								0.290	0.230	0.177
Donor								0.312	0.245	0.205
Acceptor								0.398		0.235
*r*_pred_^2^	0.695								0.864	0.797
